# Adapting a Sexual Health Intervention for Adolescents Exposed to Adversity: Feasibility Study

**DOI:** 10.2196/72782

**Published:** 2026-01-05

**Authors:** Terrinieka Powell, Bianca D Smith, Naya Moser, Olivia Kachingwe, Quiana Lewis Wallace, Asari Offiong, Andrea Hwang, Emily Davie, Ashleigh LoVette

**Affiliations:** 1 Johns Hopkins Bloomberg School of Public Health Baltimore, MD United States; 2 CareFirst BlueCross BlueShield Baltimore, MD United States; 3 School Mental Health Ontario Hamilton, ON Canada; 4 Yale University School of Public Health New Haven, CT United States; 5 Child Trends Rockville, MD United States; 6 University of Michigan School of Public Health Ann Arbor, MI United States; 7 Discovery Mood & Anxiety Program Long Beach, CA United States; 8 Gila River Health Care Sacaton, AZ United States; 9 The Ohio State University Columbus, OH United States

**Keywords:** sexual health, adolescent, adverse childhood experiences, intervention, recruitment, feasibility, engagement

## Abstract

**Background:**

Although sexual exploration is normative during adolescence, sexual activities that are unprotected and occur under the influence of substances can pose significant risks to young people. Youth exposed to adversity are among the groups most vulnerable to sexual risk-taking in adolescence. Selective interventions that consider lived experiences and the local context may help reduce sexual risk-taking among this population.

**Objective:**

This pilot study assessed the feasibility of participant recruitment and retention as well as participant engagement with an adapted version of Focus on Youth with Informed Parents and Children Together for Black youth exposed to household challenges.

**Methods:**

Participants were recruited using school and community presentations, digital flyers, and referrals. A total of 121 youth from 3 sites in Baltimore, Maryland, were screened. Participants completed 3 assessments: baseline, posttest, and 3-month follow-up. Participant enrollment, session attendance, and assessment completion were used to determine feasibility and engagement. Sexual health knowledge, pregnancy intentions, partner communication, and sexual behaviors were explored as secondary outcomes.

**Results:**

Funded by the National Institutes of Health, the data for this study were collected between January 2022 and April 2023. A total of 61 youth (aged 13-16 years) were recruited and randomized to either the intervention or the control condition (n=33 and n=28, respectively). In total, 87% (53/61) of the participants completed all 3 assessments. There was high engagement: 80% (48/61) of participants attended at least 3 sessions, and 75.2% (115/153) of after-session responses revealed they would recommend a session to a friend. Among the 18 participants who reported having any sex, all 18 (100%) abstained from alcohol use and 12 (67%) abstained from drug use before sex. The intervention group showed a significant increase in sexual health knowledge. No changes in sexual health behaviors or partner communication were observed.

**Conclusions:**

Findings suggest that recruiting, retaining, and engaging participants in the adapted Focus on Youth with Informed Parents and Children Together intervention is feasible. Additional research is needed to determine the extent to which this intervention can mitigate sexual risk-taking among youth exposed to adversity. The findings will inform the redesign of our assessments to capture additional factors that may affect sexual health behaviors.

**Trial Registration:**

ClinicalTrials.gov NCT05033821; https://clinicaltrials.gov/study/NCT05033821

## Introduction

Adverse childhood experiences (ACEs) are potentially traumatic events that occur before the age of 18 years and can have significant negative impacts on adolescents’ health and well-being [1,2]. In a meta-analysis of more than 250,000 participants, Hughes et al [3] estimated that the global prevalence of an ACE ranged from 38.8% to 59.3%, with an average of 57%. Disparities exist among youth exposed to ACEs, with Black youth being twice as likely to report at least 1 ACE compared to White youth [4]. In Baltimore, Maryland, where 79% of the youth are Black, experiences of household challenges, a subset of ACEs, are common. According to the Centers for Disease Control and Prevention’s Youth Risk Behavior Survey, among youth in Baltimore, 28% had a parent with a mental illness, 25% had a parent with a history of substance use, and 46% had a family member who had gone to prison or jail [5].

Adolescent sexual risk-taking and substance use contribute to some of the most prevalent yet preventable public health problems in the United States [6]. Adolescents have higher rates of unprotected sex and substance experimentation than adults [7-9]. Adolescent substance use is associated with increased rates of sexual intercourse, multiple sexual partners, and lower rates of condom use [10,11]. Adolescents who use substances are more likely to contract a sexually transmitted infection or become pregnant than their nonsubstance-using peers [12,13]. Findings from the 25 countries included in the Violence Against Children and Youth Surveys found that exposure to ACEs is associated with lifetime experiences of sexually transmitted diseases, unwanted pregnancies, and sexual risk-taking [14-16]. In Baltimore, 16.9% of youth reported being currently sexually active, of whom 50.4% did not use a condom during their last sexual encounter, and 20% reported using substances before sexual intercourse [5]. Baltimore youth who reported 1 household challenge were 80% more likely to report alcohol use, 78% more likely to report marijuana use, 137% more likely to report heroin use, and 231% more likely to report nonmedical use of prescription opioids [17]. The co-occurrence of these risk behaviors among adolescents warrants preventive interventions that target both [18-20].

Programs designed to reduce sexual risk-taking and related substance use may fail to reach youth today, given that most were developed and deemed effective before the year 2000 and before the integration of technology in learning, expanded vocabulary of gender identity, and comprehensive conversations about consent [21-25]. Thus, adapting evidence-based interventions to reflect current contexts may be an efficient and cost-effective strategy to address risk-taking among adolescents and build on previous effective interventions [26].

Focus on Youth with Informed Parents and Children Together (hereafter referred to as FOY+ImPACT) is a universal, group-level, Centers for Disease Control and Prevention-endorsed, evidence-based intervention that reduces sexual risk-taking and substance use behaviors among urban adolescents [27,28]. Developed in Baltimore, Maryland, it is a comprehensive community-based intervention that involves caregivers in a single session. Our research team adapted and updated the FOY+ImPACT to be delivered to youth exposed to household challenges. On the basis of formative work with families experiencing household challenges, we incorporated trauma-informed principles and gender-inclusive language, standardized the session length, and modernized the content. The process is detailed elsewhere [29]. FOY+ImPACT is a promising approach to address sexual risk-taking among Black youth exposed to household challenges. Thus, this study aimed to assess the feasibility of participant recruitment and retention, participant engagement, and the initial efficacy of the adapted program among this group.

## Methods

### Study Design

From January 2022 to April 2023, we partnered with 3 local organizations to assess the feasibility and initial efficacy of the adapted FOY+ImPACT intervention in a small, randomized trial. An online random number generator was used to randomize participants to either the control or the intervention group. Randomization occurred at the family level, allowing all eligible youth from a single family to be in the same condition and minimizing the potential for contamination of intervention effects. Participants completed electronic assessments at 3 time points: baseline, posttest, and 3-month follow-up.

### Ethical Considerations

The study was approved by the Johns Hopkins Bloomberg School of Public Health Institutional Review Board in Baltimore, Maryland (approval 17282). Interested youth were required to complete a participant interest form that included their name, as well as their caregiver’s name and contact information. A study team member contacted the caregivers to inform them of the study and assist with completing a screener to determine the youth’s eligibility. A study team member reviewed the purpose and procedures of the study, as outlined in the parental permission form. The permission form was emailed and texted to caregivers via Qualtrics (Qualtrics LLC) to obtain signatures. Once permission was granted, all eligible youth in the family were assigned a participant ID. Oral assent was obtained from all youth prior to completion of the baseline survey. Caregivers and participants were informed that they could stop participating at any point without consequences. The trial was registered at ClinicalTrials.gov (NCT #05033821). Participants were compensated US $25 for each session they attended and each assessment they completed. All participants received a participant identification number and data were deidentified before analysis.

### Recruitment and Enrollment

Youth enrollment occurred 1 month before the start of the intervention. To be eligible, participants had to be aged 13-16 years, identify as Black or African American, and have experienced 1 or more household challenges. Direct youth recruitment occurred when the study team members invited families to participate via presentations at each organization. Participants were also recruited indirectly through referrals from organizational staff and enrolled participants. Recruitment materials and presentations did not include household challenges as an eligibility criterion, thereby reducing the risk of stigma associated with participation.

### Curriculum Delivery and Content

Both intervention and control group sessions occurred twice a week, in person, during nonschool hours (eg, summer or after school). No more than 10 youth were allowed to participate in each group. Sessions were co-facilitated by 2 study team members.

### Adapted FOY+ImPACT

Consistent with the original version of FOY+ImPACT, the adapted version had 2 components: Focus on Youth (FOY) and ImPACT. The FOY component was an 8-session intervention emphasizing the core components of establishing a strong support network, decision-making, goal setting, communication, and negotiation. This adapted curriculum incorporated trauma-informed principles and gender-inclusive language, standardized session length to 100 minutes, and updated age-appropriate content for the priority population. The ImPACT component was a single-session intervention for youth and caregivers, in which families received information about sexual health topics and practiced communication skills. This single session was held via Zoom (Zoom Video Communications) before the third FOY session.

### Youth in the Media

We used a time-matched control group to reduce temporal bias and control for potential unmeasured confounds; control group participants participated in a time-matched program called Youth in the Media (YM). Delivered in 8 in-person, 100-minute sessions concurrently with FOY sessions, the YM program was designed to (1) increase participants’ awareness of and access to the different types of media and free resources available at their local libraries, (2) discuss different types of media that center on stories and experiences of Black youth, and (3) expose participants to careers in media. Sessions covered a range of media types, including sports broadcasting, spoken word, blogging, and comic books. Each session opened with a theme-related icebreaker and closed with a review of key points and a lesson-related practice activity. There was no caregiver component for the YM program.

### Measures

#### Primary Outcomes: Feasibility and Engagement

##### Feasibility

Process measures were assessed through interest forms and screening logs. We defined recruitment success as the ability to recruit at least 10 youth per cycle. Retention was assessed by the proportion of youth who completed the posttest and the 3-month follow-up. We defined retention success as more than 80% of the sample being retained at the posttest and 3-month follow-up.

##### Engagement

Engagement was assessed in 2 ways. First, dosage (ie, the number of sessions the participant attended) was used to assess engagement. We defined high-dosage success as youth attending ≥5 sessions, moderate dosage as attending at least 3 sessions, and low dosage as participants attending ≤2 sessions. Second, we administered feedback surveys at the end of each session. These surveys asked if participants would invite a friend to a session (ie, yes, no, or maybe) and “What, if anything, could have made the session better?” The latter was an open-ended question.

#### Secondary Outcomes: Knowledge, Attitudes, Communication, and Behaviors

##### Sexual Health Knowledge

A total of 15 items assessed sexual health knowledge. The questions covered topics related to sexually transmitted infections, contraceptives, and contraceptive use behaviors. All questions were true or false statements. Each correct response was given 1 point, whereas an incorrect response was scored 0. Scores were summed up, and the maximum score was 15. Cronbach α was 0.54 at baseline, 0.86 at posttest, and 0.69 at the 3-month follow-up.

##### Pregnancy Attitudes

A total of 5 items assessed attitudes about pregnancy during adolescence. Participants were asked to indicate their level of agreement with general statements about pregnancy (eg, “I am okay with being pregnant now” and “I am ready to be a parent”). Two questions were reverse-coded (eg, “My family would be really upset if I got pregnant or got someone pregnant as a teen” and “I would be really upset if I got pregnant or got someone pregnant”). All questions were measured using a 4-point Likert scale. Scores were summed up and averaged. A higher score indicated fewer negative attitudes about pregnancy during adolescence. Cronbach α was 0.40 at baseline, 0.66 at posttest, and 0.63 at the 3-month follow-up.

##### Partner Communication

A total of 4 items were used to create a scale of perceptions about communicating with a partner about sexual health. Participants were asked to indicate their level of agreement with statements about communicating about condom use (eg, “How difficult would it be for you to ask a partner to use a condom, even if it might make them think you have a sexually transmitted disease?” and “How difficult would it be for you to start requiring a partner to use a condom, even though you haven’t used one in the past?”). All questions were measured using a 4-point Likert scale (1=not difficult at all; 2=not very difficult; 3=somewhat difficult; and 4=very difficult). The average of the items was used to capture partner communication. A higher score indicated higher levels of difficulty in communicating with a partner about condom use. Cronbach α was 0.80 at baseline, 0.84 at posttest, and 0.83 at the 3-month follow-up.

##### Behaviors

Sexual health behaviors were assessed in 2 ways. First, participants responded to a question about whether they had ever had sex or had sex in the past 3 months. If they reported ever having sex, they were asked about alcohol, drugs, and contraceptive use during their last sex. Participants responded “yes” or “no.”

### Data Analysis

Descriptive statistics were calculated for demographics, feasibility of recruitment and retention, and sexual health knowledge, pregnancy attitudes, partner communication, and behaviors. Open-ended responses were analyzed deductively using a qualitative content analytic approach [30]. Using this approach, the study team reviewed and summed the responses to identify the major themes. Two-tailed paired *t* tests were used to compare the means of knowledge, pregnancy attitudes, and partner communication at each time point in the study. A mixed repeated measures ANOVA was used to test whether the intervention influenced changes in knowledge, pregnancy attitudes, and partner communication over time. Chi-square tests assessed changes in behavior over time and by group assignment. All statistical analyses were conducted using Stata (version 18.0; StataCorp).

## Results

### Feasibility and Engagement

**Figure 1:**
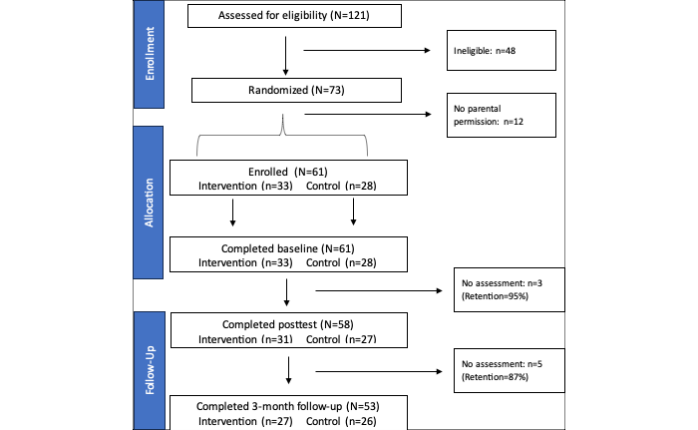
Participant flowchart for the pilot study.

Figure 1 displays the participant flowchart for this study. More than 150 youth or parents completed an interest form to be screened for eligibility (N=121). In total, 39.6% (48/121) of the interested youths were ineligible because they did not have a caregiver with a history of substance use, mental health issues, or incarceration. Among the 73 eligible participants, 12 (16%) did not return their parental permission forms after at least 5 attempts from the study team. Ultimately, 61 Black youth (aged 13-16 years) exposed to household challenges were enrolled in the study and randomized to a condition. A total of 87% (53/61) of the participants completed all 3 assessments, and 62% (38/61) attended at least 5 sessions (high dosage). We received 153 feedback survey responses across all sessions. Of the 153 responses, 115 (75.2%) said “yes” when asked if they would invite a friend to the session. When asked about what could be done to improve the sessions, 61 participants provided 111 responses across all sessions. The most frequent responses (75/111, 67.6%) were “nothing, none, or nope,” coupled with comments such as “it was really good,” “it was perfect,” and “I was satisfied.”

### Knowledge, Attitudes, Communication, and Behaviors

We used a moderate dosage to assess the initial efficacy. A total of 20% (12/61) of the participants were excluded from the analysis for initial efficacy due to low dosage (eg, attending ≤2 sessions). Thus, the analytic sample size was 80% (49/61) of Black youth of the initial sample who attended an average of 6 sessions. The average age of our sample was 15 years, with those in the intervention group being significantly younger than those in the control group (*P*=.015). There were more male participants (26/49, 53%) than female participants (20/49, 41%), or those who identified with other gender identities (3/49, 6%). Having an incarcerated parent was the most common household challenge (22/49, 46%), followed by having a parent with a mental illness (15/49, 31%), and a parent with a history of substance use (13/49, 27%). Table 1 presents the detailed demographic statistics for the sample.

Table 2 displays the results of the paired *t* test at each time point. The means of sexual health knowledge and pregnancy attitude scores were similar across the groups. At baseline, there were significant differences in partner communication beliefs, with the intervention group finding it more difficult to communicate with a partner about sexual health topics compared to the control group. Posttest and 3-month follow-up results showed no significant differences between the groups at each time point.

Table 3 displays the results from the repeated mixed ANOVA comparing time and group assignment across all domains. There was a significant interaction between time and study group assignment for knowledge scores (*F*_2,47_=3.27; *P*=.04). The effect size for changes in knowledge over time by group assignment was moderate (η^2^=0.08). Post hoc comparisons showed that trends in the mean knowledge scores in the intervention group increased over time but these results were not statistically significant (*P*=.07). However, there were significant changes in knowledge within the control group over time, where knowledge scores decreased from baseline to the posttest (*P*<.001) and increased from the posttest to the 3-month follow-up (*P*=.03). There were also significant differences in pregnancy attitudes over time but not by group assignment. Youth in both groups had fewer negative attitudes about pregnancy during adolescence from baseline to the 3-month follow-up (*F*_2,47_=28.22; *P*<.001). The effect size was large for pregnancy attitudes (η^2^=0.40), which indicated a large and meaningful effect. No significant findings were observed regarding partner communication.

At baseline, most participants (30/48, 63%) reported never having any type of sex. Among those who reported having any type of sex, 100% (18/18) abstained from alcohol use before sex, and 67% (12/18) abstained from drug use before sex. More than half (8/15, 53%) of the sexually active youth who responded to questions about contraception reported the use of birth control during their last sexual encounter. The intervention participants found it significantly more difficult to communicate with a partner about contraceptive use than the control group at baseline (*P*=.03). Trends suggest that the intervention group reported more contraceptive use in the last sex than the control group, but these results were not statistically significant (*P*=.07). However, the posttest and 3-month follow-up results showed no significant differences in behaviors between the groups.

**Table 1:** Baseline demographic characteristics.

Variable			Overall (N=49)		Control (n=21)		Intervention (n=28)		*P* value (control vs intervention)	
Age (years), mean (SD)			15.10 (0.82)		15.43 (0.60)		14.86 (0.89)		*.02* ^a^	
Attendance, mean (SD)			6.04 (1.68)		5.95 (1.77)		6.11 (1.64)		.75	
**Gender, n (%)**										.44
	Female	20 (41)		11 (52)		9 (32)				
	Male	26 (53)		9 (43)		17 (61)				
	Nonbinary	2 (4)		1 (5)		1 (4)				
	Other (gender-fluid)	1 (2)		0 (0)		1 (4)				
Sexual health knowledge, mean (SD)			11.48 (1.71)		11.9 (1.80)		11.18 (1.61)		.15	
Pregnancy attitudes, mean (SD)			1.73 (0.39)		1.69 (0.40)		1.75 (0.38)		.63	
Partner communication, mean (SD)			2.02 (0.89)		1.70 (0.76)		2.26 (0.92)		*.03*	
**Have had any type of sex** **(vaginal, oral, or anal), n (%)**										.60
	Yes	18 (38)		7 (33)		11 (41)				
	No	30 (63)		14 (67)		16 (59)				
**Used alcohol before sex,** **n (%)**										—^b^
	Yes	0 (0)		0 (0)		0 (0)				
	No	18 (100)		6 (100)		12 (100)				
**Used drugs before sex,** **n (%)**										>.99
	Yes	6 (33)		2 (33)		4 (33)				
	No	12 (67)		4 (67)		8 (67)				
**Current sex (sex in the past 3 mo),** **n (%)**										.67
	Yes	9 (60)		4 (67)		5 (56)				
	No	6 (40)		2 (33)		4 (44)				
**Used contraception at last sex,** **n (%)**										.07
	Yes	8 (53)		1 (20)		7 (70)				
	No	7 (47)		4 (80)		3 (30)				

^a^Italics indicate a statistically significant difference.

^b^Not applicable.

**Table 2:** Two-tailed paired *t* tests for knowledge, attitudes, and communication (N=49).

	Baseline, mean (SD)				Posttest, mean (SD)				3-mo follow-up, mean (SD)		
	Combined	Control	Intervention	Combined		Control	Intervention	Combined		Control	Intervention
Sexual health knowledge	11.48 (1.71)	11.9 (1.80)	11.18 (1.61)	10.74 (2.87)		10.06 (2.92)	11.24 (2.79)	11.44 (2.70)		11.22 (2.67)	11.6 (2.77)
Pregnancy attitudes	1.73 (0.39)	1.69 (0.40)	1.75 (0.38)	1.51 (0.53)		1.55 (0.54)	1.47 (0.53)	2.14 (0.46)		2.08 (0.50)	2.18 (0.44)
Partner communication	2.02 (0.89)	1.70 (0.76)	2.26 (0.92)	2.33 (0.96)		2.31 (1.02)	2.35 (0.92)	2.23 (1.0)		1.92 (1.02)	2.46 (0.93)

**Table 3:** Repeated mixed ANOVA results (N=49).

			*F* test (*df*)		*P* value		Partial η² (effect size)
**Sexual health knowledge**							
	Time	2.93 (2, 47)		.06		0.07	
	Assignment	0.31 (1, 48)		.58		0.01	
	Interaction (time x assignment)	3.27 (2, 47)		*.04* ^a^		0.08	
**Pregnancy attitudes**							
	Time	28.22 (2, 47)		*<.001*		0.40	
	Assignment	0.00 (1, 48)		.96		0.00	
	Interaction (time x assignment)	0.54 (2, 47)		.59		0.01	
**Partner communication**							
	Time	2.33 (2, 47)		.10		0.05	
	Assignment	3.38 (1, 48)		.07		0.07	
	Interaction (time x assignment)	1.19 (2, 47)		.31		0.03	

^a^Italics indicate a statistically significant difference.

## Discussion

This study aimed to assess the feasibility of participant recruitment and retention, as well as participant engagement with an adapted program for youth exposed to household challenges. Exposure to household challenges can affect youth behaviors and their options for healthy decision-making. Although all youth were exposed to 1 or more household challenges, among those who were sexually active, many reported using protection and abstaining from substance use during sex. The adapted intervention improved the sexual health knowledge of the intervention participants. Both the intervention and control group participants also became less negative toward pregnancy during adolescence over time. However, the groups did not differ in partner communication or sexual health behaviors over time.

While many teen pregnancies are unplanned and unintended, some may be planned and intended [31,32]. One study found that nearly 30% of adolescent females desired to be pregnant within 5 years [33]. Furthermore, as young people age and have prolonged exposure to household challenges, negative views of pregnancy may dissipate, given the perceived rewards of filling emotional bonds [34]. Thus, the tendency of study participants to become less negative may be a function of their maturity and environment. Future studies should consider assessing and offering tailored resources to young people who desire pregnancy and those who do not. Using this approach, findings related to sexual behaviors can be understood in the context of the individual reproductive health plans of young people.

The lack of group differences in partner communication and sexual health behaviors may best be attributed to the low baseline rates of sexual risk-taking and low dosage. More than one-third of the participants received a low or moderate dose of the intervention. Dosage significantly affects intervention outcomes [35-37]. Thus, higher exposure may have a more positive impact on partner communication. In addition, although 38% (18/48) of the youth reported having sex at baseline, nearly all were doing so responsibly (with protection and without substances). Therefore, the opportunities to observe changes over time were minimal.

Methodological limitations of this pilot study include a small sample size, limited measures, low internal reliability of measures, and no long-term follow-ups. Although the sample size and follow-up window were dictated by funding, improvements in the measure can be more easily addressed in future research through the refinement of measures and the integration of motivational components into the intervention. The extent of missingness and bias across our measures may have contributed to the observed results. Other factors that we did not capture may have influenced participant attendance and outcomes. According to behavior change theories [38-41], increasing knowledge is an essential precursor to changing behaviors, but it is not sufficient. Motivation, self-efficacy, fear, the desire for status and respect, and external factors also play a significant role in behavior change [42,43]. Study groups did not differ by most demographic factors or initial scores on outcome measures. In future studies, we will extend the follow-up period and redesign our questionnaire to capture additional factors that may impact sexual health behaviors. Additionally, we will explore other strategies to ensure that students complete all parts of the survey, including reading the survey to students or offering the option to take it on multiple days to prevent survey fatigue.

Despite these limitations, the study findings suggest that the adapted FOY+ImPACT was feasible and engaging. However, additional research is needed to determine the extent to which it can mitigate sexual risk-taking among youth exposed to adversity. For youth exposed to household challenges, parents may not be readily available. The adapted FOY+ImPACT intervention offered information and strategies for youth to connect with their parents, peers, partners, and other trusted adults to promote healthy sexual health habits. A growing body of research has demonstrated the value of tailoring interventions for new groups as a strategy to support population health and extend the impact of evidence-based interventions [26,29,44,45]. Study findings also provide additional evidence of the complexity of adolescent sexual health decision-making, especially among youth exposed to household challenges. For example, although knowledge increased and pregnancy attitudes became less negative, no significant behavioral changes were observed. Thus, additional research is needed to understand what will work, for whom, and under what circumstances to reduce sexual health risk-taking.

## Abbreviations

ACE: adverse childhood experience
FOY: Focus on Youth
FOY+ImPACT: Focus on Youth with Informed Parents and Children Together
YM: Youth in the Media

## Data Availability

The datasets generated or analyzed during this study are not publicly available because participants did not consent for their data to be shared publicly.
